# Anthracycline-free neoadjuvant therapy induces pathological complete responses by exploiting immune proficiency in HER2+ breast cancer patients

**DOI:** 10.1186/1471-2407-14-954

**Published:** 2014-12-15

**Authors:** Gianmaria Miolo, Elena Muraro, Debora Martorelli, Davide Lombardi, Simona Scalone, Simon Spazzapan, Samuele Massarut, Tiziana Perin, Elda Viel, Elisa Comaro, Renato Talamini, Ettore Bidoli, Elisa Turchet, Diana Crivellari, Riccardo Dolcetti

**Affiliations:** Department of Medical Oncology, National Cancer Institute, Via F. Gallini 2, 33081 Aviano, PN Italy; Cancer Bio-Immunotherapy Unit, Department of Medical Oncology, C.R.O, National Cancer Institute, Via F. Gallini 2, 33081 Aviano, PN Italy; Division of Breast Surgical Oncology, National Cancer Institute, Via F. Gallini 2, 33081 Aviano, PN Italy; Pathology Unit, National Cancer Institute, Via F. Gallini 2, 33081 Aviano, PN Italy; Cardiology, National Cancer Institute, Via F. Gallini 2, 33081 Aviano, PN Italy; Unit of Epidemiology and Biostatistics, National Cancer Institute, Via F. Gallini 2, 33081 Aviano, PN Italy; Scientific Direction, CRO Aviano, IRCCS, National Cancer Institute, Via F. Gallini 2, 33081 Aviano, PN Italy

**Keywords:** Breast cancer, Neoadjuvant chemotherapy, HER2, Immune response, Trastuzumab

## Abstract

**Background:**

Neoadjuvant Chemotherapy (NC) including trastuzumab induces a high rate of pathological Complete Responses (pCR) in patients with locally advanced HER2-overexpressing Breast Cancer (BC), but is penalized by a severe cardiotoxicity when combined with anthracyclines. A phase II study was designed to assess whether an anthracycline-free NC regimen based on the early addition of trastuzumab to paclitaxel may increase the pCR rate without inducing severe cardiotoxicity in patients with locally advanced HER2-overexpressing BC. Immunomonitoring was performed to assess the contribution of patients’ immunological background to the induction of clinical responses.

**Methods:**

Stage II-III HER2-positive BC patients received 24 weeks paclitaxel and trastuzumab NC, followed by 1 year adjuvant trastuzumab ± hormonal and/or radio-therapy. Assessment of pCR rate was the primary endpoint. A group of HER2-negative BC patients treated with neoadjuvant taxanes and anthracyclines was included. Serum levels of 10 cytokines and the efficiency of trastuzumab-mediated antibody-dependent cell cytotoxicity (ADCC) were monitored *in vitro* every 3 months.

**Results:**

From July 2006 to February 2013, we enrolled 109 patients including 46 evaluable HER2-positive cases. A pCR rate of 50% was reached and no severe cardiotoxicity occurred. Serum cytokine profiling revealed only an IL-10 decrease (*P* = 0.02) in patients achieving a partial response, while HER2-negative patients disclosed marked cytokines changes. Compared to the unfavourable F/F genotype, patients carrying the V allele in the FcγRIIIa-158 polymorphism showed a higher efficacy of trastuzumab-ADCC throughout treatment (*P* ≤0.05).

**Conclusions:**

In the absence of anthracyclines, trastuzumab and paclitaxel induced a high rate of pCR, exploiting the synergy between the immunomodulating properties of these drugs and the retained immunological proficiency of patients with HER2-overexpressing BC.

**Trial registration:**

Trial registration number: NCT02307227, registered on ClinicalTrials.gov (http://www.clinicaltrials.gov, November 26, 2014).

## Background

Trastuzumab-containing neoadjuvant chemotherapy (NC) has significantly changed the standard of care for locally advanced HER2-positive breast cancer (BC) patients, demonstrating the ability to increase pathological Complete Response (pCR) rates up to 65% [1]. Despite their efficacy, however, clinically active NC regimens containing both anthracyclines and trastuzumab are often burdened by cardiotoxicity as a limiting side effect, which still constitutes an open issue [2, 3]. Conversely, in the NOAH trial, despite the concurrent use of doxorubicin, paclitaxel and trastuzumab, incidence of symptomatic cardiac failure was low (<2%) [3, 4]; however, long-term data on cardiotoxicity are still lacking.

Available evidence indicates that HER2-positive BC patients achieving pCR after NC have better relapse-free and survival rates compared with those showing partial responses [5]. Nevertheless, only limited information is available on the possible predictors of pCR by NC, an issue that is crucial to identify patients who may benefit from additional treatment. Accumulating data support a relevant role of host immune responses in mediating the clinical efficacy of NC, particularly when using drugs with immunomodulating properties, such as taxanes [6]. Immune activation parameters were significantly associated with the induction of pCR in HER2-overexpressing BC patients treated with NC [7]. In particular, the efficacy of trastuzumab in mediating antibody-dependent cell cytotoxicity (ADCC) [8] is the result of the complex engagement of different factors, including activating and inhibitory Fc receptor gene polymorphisms, which have been correlated with objective response rate (ORR) in BC patients [9, 10]. Interestingly, ADCC efficiency and the number of circulating natural killer (NK) cells were related to trastuzumab clinical efficacy in primary operable BC patients [11]. Recent evidence also suggested that cytokine serum levels might provide useful correlates of the response to NC [12].

This study was designed to assess whether the addition of trastuzumab in an early phase in combination with taxanes may increase NC-induced pCR rates in patients with locally advanced HER2-overexpressing BC without the burden of cardiac dysfunction related to anthracyclines. The rationale of this approach is based on the consideration that patients usually receive ≥3 cycles of anthracyclines before starting trastuzumab, with a significant delay in the use of the most active drug in this setting. Moreover, several immune parameters were investigated throughout NC to assess the contribution of the immunological background of treated patients to the induction of clinical responses. The setting of HER2-overexpressing BC is particularly suited to carry out this analysis, considering our previous results indicating that these patients, at diagnosis, retain an unaltered immune proficiency that can be exploited by NC drugs such as trastuzumab or taxanes [13].

## Methods

### Study design and participants

This was a single arm, phase II mono-institutional study. Patients with HER2-positive locally-advanced BC received neoadjuvant weekly paclitaxel (80 mg/m^2^ on day 1,8,15,22 repeated every 4 weeks [TP]) concurrently with trastuzumab (loading dose 4 mg/kg intravenously, then 2 mg/kg weekly) for 3 cycles, followed by evaluation and, in case of clinical response, 3 more cycles were administered to obtain pCR. After NC completion, patients underwent primary surgery (mastectomy or conservative treatment) and axillary node dissection. After surgery, adjuvant paclitaxel and trastuzumab was continued for 3 cycles and trastuzumab alone was administered every 3 weeks for 1 year. Radiation and/or hormonal therapy was performed if indicated. As controls for immune parameters, a parallel HER2-negative BC group was prospectively included and treated with a NC regimen (8 cycles of docetaxel [75 mg/m^2^] and concomitant epirubicin [90 mg/m^2^] every 3 weeks [ED]).

The primary outcome was to determine the pCR rate. pCR was defined as no evidence of microscopic residual invasive cancer, both in breast and ipsilateral axillary lymph nodes, or residual carcinoma *in situ* in the absence of invasive breast cancer [14]. Secondary endpoints were ORR, disease-free survival (DFS), overall survival (OS), and toxicity. This study (CRO-18-2006) was conducted according to the ethical principles of the Declaration of Helsinki and approved by the local Ethical Committee (Comitato Etico Indipendente del CRO di Aviano, may 29, 2006). Written informed consent was obtained from all patients.

Eligibility criteria were: age ≤ 70 years; histologically confirmed locally advanced BC (UICC stage II-III, non-inflammatory) evaluated for *HER2/neu* status; Eastern Cooperative Oncology Group performance status of 0 or 1; baseline left ventricular ejection fraction (LVEF) >50% measured by ultrasonography; adequate organ function (bone marrow function: neutrophils ≥2.0x10^9^/L, platelets ≥120x10^9^/L; liver function: serum bilirubin <1.5 times the upper normal limit [UNL], transaminases <2.5 times UNL, alkaline phosphatase ≤2.5 times UNL, serum creatinine <1.5 times UNL) and measurable disease according to the Response Evaluation Criteria in Solid Tumors (RECIST). Exclusion criteria were brain metastases, previous chemotherapy or hormonal therapy, prior myocardial infarction or uncontrolled arrhythmia or angina pectoris or other serious medical conditions or psychiatric syndromes; concurrent malignancy other than non-melanoma skin cancer, or *in situ* cervix carcinoma.

Baseline evaluation included a physical examination (including evaluation of vital signs and performance status), laboratory tests (haematology and clinical chemistry, CA15.3), diagnostic breast imaging (mammogram, ultrasound, and magnetic resonance imaging), abdominal ultrasound, bone scintigraphy and LVEF measurement by echocardiography. Metallic markers were placed into the breast under ultrasound examination before chemotherapy. Instrumental evaluation was performed at baseline and every 12 weeks. RECIST criteria were used to evaluate the response. Adverse events were graded according to the National Cancer Institute Common Toxicity Criteria version 3, and the worst toxicity per cycle was recorded. LVEF was evaluated every two cycles and cardiac events were graded according to NYHA.

Surgical evaluation was planned at baseline and at the end of NC. Patients obtaining a clinical complete response or appropriate candidates for breast conservation therapy (BCT) were offered quadrantectomy, whereas patients not eligible for BCT underwent total mastectomy. Patients with clinically negative node underwent a sentinel lymph node biopsy and those who had positive nodes underwent axillary lymph node dissection. Patients treated with a segmental mastectomy received whole breast irradiation after the end of chemotherapy. Radiation treatment of the chest wall and draining lymphatics was performed in patients with stage III disease and with ≥4 positive lymph nodes.

The aim of this phase II clinical trial was to show an increase of a further 20% in the pCR rate (≥40%). The projected pCR rate with treatment without trastuzumab was estimated to be ≈ 20% based on previous experience with similar chemotherapy [15]. Simon’s method was used to calculate sample size. Accrual of 46 patients was planned considering an 80% of power to detect a 20% difference (two-sided type I error = 0.05). The Chi-square test and Fisher’s exact test were used for qualitative parameters. Statistical differences within quantitative parameters were determined by Wilcoxon rank-test (non-parametric test) for two samples. Results were considered statistically significant when *P* ≤ 0.05 (two-sided). Statistical analyses were performed with the SAS software (version 9.0).

### Immunohistochemistry

Clone 6 F11 and clone 1E2 (Ventana Medical Systems, Inc., Tucson, AZ) were used for estrogen and progesterone receptor evaluation, respectively. With the Allred score, the proportion score and the intensity score are assessed in six and four grades, respectively 0-5 and 0-3, then the total score is assessed in eight grades (0 and 2-8) [16]. Tumors with an immunohistochemistry (IHC) total score of 3 were reported as positive. A score index of 0, 1, 2, and 3 was used, corresponding to negative, weak, moderate, and strong staining intensity, respectively, and the percentage of positive cells at each intensity was estimated subjectively. *HER2/neu* scores of 0-1 were considered negative, whereas a score of 3 was reported as positive (DAKO). Chromogenic *in-situ* hybridization or fluorescence *in-situ* hybridization analyses were performed in cases with IHC total score of 2.

### Blood sample collection

Heparinised blood and sera were collected from each patient at diagnosis and throughout NC, after 12 and 24 weeks of treatment. Peripheral blood mononuclear cells (PBMCs) were freshly isolated from heparinised blood of patients by Ficoll-Hypaque gradient (Lymphoprep, Fresenius Kabi Norge Halden, Norway) using standard procedures and viably frozen at -180°C until use. Serum samples were obtained with blood centrifugation at 2,100 rpm and maintained at -80°C.

### Serum cytokine detection

Levels of interleukin (IL)-1α, IL-1β, IL-2, IL-6, IL-8, IL-10, IL-12p70, tumor-necrosis factor-α (TNF-α), and granulocyte macrophage colony-stimulating factor (GM-CSF) were evaluated using the SearchLight® multiplex arrays (Food and Drug Administration approved, Aushon Biosystems, TEMA Ricerca, Bologna, Italy) according to the manufacturer’s instructions. Briefly, custom human 8-plexarray and human 1-plexarray (for GM-CSF detection) with pre-spotted cytokine-specific antibodies were used. Standards or pre-diluted samples were added in duplicate and, after 1 hour of incubation at room temperature and 3 washes, biotinylated antibody reagent was added to each well. After 30 minutes incubation at room temperature and 3 washes, block solution was added to stabilize the signal. The addition of Streptavidin-HRP Reagent and SuperSignal® Substrate, and the acquisition of luminescent signal with a cooled Charge Coupled Device camera, together with data analysis and processing, were performed by TEMA Ricerca laboratories’ customer service (Bologna, Italy). Transforming growth factor (TGF)-β1 serum levels were assessed through ELISA (DRG Instruments GmbH, Marburg, Germany) under manufacturer’s instructions. Pre-diluted samples and standards underwent appropriate acidification and neutralization before testing. Briefly, pre-treated standards, controls and samples were dispensed into wells in duplicate and plates were incubated overnight at 4°C. After 3 washes, antiserum was added to wells and incubated for 120 minutes at room temperature, plate was rinsed 3 times and anti-mouse biotin (enzyme conjugate) was dispensed and incubated for 45 minutes. After 3 washes, enzyme complex was added to wells, plates were incubated 45 minutes and washed 3 times. After the addition of substrate solution for 15 minutes, the reaction was stopped and the absorbance at 450 ± 10 nm was determined with a microtiter plate reader (Bio-Tek Instruments, Winooski, VT, USA).

### ADCC assay

Trastuzumab-dependent ADCC efficiency was evaluated in a Calcein release assay, using the Her2/*neu*-overexpressing breast cancer cell line MDA-MB453, authenticated by fingerprinting in November 2013 (Power Plex 1.2, Promega, Madison, WI, USA) as tumor model target cells, and patients’ PBMCs as effectors. The cell line was cultured in DMEM (Sigma), containing 2 mM L-glutamine, 10% FBS (Gibco®, Life Technology, Grand Island, New York, USA), 100 μg/ml streptomycin and 100 IU/ml penicillin (Sigma-Aldrich, St. Louis, MO, USA), at 37°C in 5% of CO_2_. One million target cells in exponential growth was labelled with 7.5 μM Calcein-AM (Molecular Probes, Eugene, Oregon, USA) for 30 minutes at 37°C, washed 3 times, then incubated with trastuzumab antibody (20 μg/ml; Roche, Basel, Switzerland) 1 h in ice. Without washing the persistence of soluble antibody, 1 × 10^4^ labelled target cells per well were seeded into 96-well U-bottom plates. Experiments were conducted in triplicates at 2 effector (PBMCs):target ratios of 30:1 and 15:1, in 200 μl of HBSS containing 5% FCS. After 4 h at 37°C and 5% CO_2_ the release of Calcein (excitation = 485 nm; emission = 530 nm) was measured with a fluorescence plate reader (SpectraFluorPlus, Tecan, Männedorf, Switzerland). Maximal and spontaneous Calcein release values were obtained by adding either 100 μl Lysis buffer (NaBO_3_ 0.025 M, Triton X-100 0.1%, pH9) or HBSS, to wells containing 1 × 10^4^ labelled target cells. The percentage of lysis was calculated according to the standard formula = 100 × (experimental release – spontaneous release)/(maximal release – spontaneous release). The percentage of lysis was normalized for 10,000 NK cells using the following formula = (percentage of lysis * 10^4^)/(Effector:Target ratio * target cell n° in an experimental well * NK percentage in PBMCs) [10].

### Flow cytometry

The following fluorescent-conjugated monoclonal antibodies were used: α-CD3 phycoerythrin-texas red (ECD; mouse IgG1, clone UCHT1), and α-CD16 FITC (mouse IgG1, 3G8), from Beckman Coulter (Fullerton, CA, USA); α-CD56 phycoerythrin (PE; mouse IgG1 k, B159) purchased from BD Biosciences (Becton Dickinson, Franklin Lakes, NJ, USA). Properly labelled isotypic antibodies were used as negative controls. All antibodies were used in an appropriate volume of 10% rabbit serum (Dako, Glosdrup, Denmark) and PBS (Biomerieux, Marcy l’Etoile, France) to reduce unspecific signal. At least 10 × 10^4^ cells were acquired. Flow cytometry analysis was performed with a Cytomics FC500 (Beckman Coulter, Fullerton, CA, USA) and data were analyzed with CXP software (Beckman Coulter, Fullerton, CA, USA).

### Analysis of FcγRIIIa, FcγRIIa, and FcγRIIb polymorphisms

Genomic DNA was purified using DNA extraction kit (EZ1 DNA Blood 350 μl kit, Qiagen, Valencia, CA) from blood samples obtained at diagnosis from all patients. FcγR locus genotyping was performed on the genomic DNA of 37 patients by polymerase chain reaction (PCR) followed by direct sequencing in both forward and reverse directions, focusing on the hot spots of SNPs at FcγRIIa-131, FcγRIIIa-158, and FcγRIIb-187. All PCR reactions were conducted with 250 ng of DNA, 10pmol of each primer, 1.5 mM MgCl_2_, 10 mM dNTPs, and 2.5 unit of Taq DNA polymerase (all reagents purchased from Promega) in a 50 μl reaction volume. The FcγRIIIa-158 V/F polymorphism was investigated through a nested PCR first using the forward primer 5′-TTGAAGGCCATGCTCAGTAAT-3′ and the reverse primer 5′-AGGCTGGTGCTACAGAACCTA-3′ to amplify a fragment of 1699 bp. The PCR reaction was started at 95°C for 5 minutes, followed by 35 cycles of denaturing at 94°C for 30 seconds, annealing at 68°C for 90 seconds, and extension at 72°C for 90 seconds, with a final extension at 72°C for 10 minutes. PCR products were diluted 1:10 and 1 μl was used for the nested PCR employing the following primers: forward 5′-TTACAGAATGGCAAAGGCAG-3′, reverse 5′-TCTCCTCCCAACTCAACTTCC-3′. The PCR reaction was performed starting at 95°C for 5 minutes, and subsequent 35 cycles of denaturing at 94°C for 30 seconds, annealing at 65°C for 45 seconds, extension at 72°C for 60 seconds, and a final extension for 10 minutes at 72°C. The PCR product of 238 bp was purified and directly sequenced using the BigDye Terminator sequencing kit and an ABI Prism 3100 sequencer (both from Applied Biosystems, Foster City, CA). The FcγRIIa-131H/R polymorphisms was analysed through a single PCR followed by sequencing using the forward primer 5′-CTGGTCAAGGTCACATTCTTC-3′ and the reverse 5′-CAATTTTGCTGCTATGGGC-3′, performing 35 cycles of denaturing at 94°C for 30 seconds, annealing at 50°C for 30 seconds, extension at 72°C for 30 seconds, and a final extension for 10 minutes at 72°C amplifying a 277 bp fragment [17]. Finally, the FcγRIIb-187I/T polymorphism was investigated in PCR and then sequenced using the forward 5′-CTCTGTTCCTGCCTGCTCACA-3′ and the reverse primer 5′-CTGGCAATGTCTGGGGTTAGG-3′, obtaining a 430 bp fragment from a PCR reaction of 35 cycles of denaturing at 94°C for 30 seconds, annealing at 60°C for 45 seconds, extension at 72°C for 60 seconds.

### Statistical methods for immunological studies

The Student’s t test for two tailed distributions and paired data was used for the statistical analysis of cytokine serum levels and NK cells percentage variations during NC, comparing data obtained at diagnosis with data measured respectively after 12 and 24 weeks of treatment. The non-parametric Kruskal-Wallis test for the comparison of three independent samples was employed to investigate the ADCC lysis (absolute and normalized) percentages classified by the FcγRIIIa158 polimorphism (genotypes V/V, V/F, and F/F). The non-parametric Wilcoxon test for two independent samples was applied to compare ADCC lysis (absolute and normalized) percentages of V carriers with those of F/F individuals. Data obtained from multiple independent experiments were expressed as mean and standard deviation. Differences were considered statistically significant when P ≤ 0.05.

## Results

### Clinical response

From July 2006 to February 2013, a total of 109 patients were enrolled (Table 1). Three patients were excluded due to discordant pre- and post-operative HER2 status. In the HER2-negative group, one patient discontinued the trial due to adverse events (nausea-vomiting G4, diarrhoea G3, and neutropenic fever G4 on cycle 1) and one was lost on the post-surgical follow-up.

**Table 1:** **Patient characteristics at baseline**

Characteristics	Trastuzumab (T) + paclitaxel (P) (n = 46)	Epirubicin (E) + docetaxel (D) (n = 58)
Median age, years	46	46
Range	23 - 70	27 - 69
Hormone receptor status		
ER+ and PgR+	13	33
ER+ and PgR-	9	9
ER- and PgR-	21	15
ER- and PgR+	3	1
HER2/neu		
0-1		47
2+		11
CISH/FISH not amplified	/	11
CISH/FISH amplified	/	/
3+	46	/
Stage distribution		
IIA	6	20
IIB	27	27
IIIA	12	10
IIIB	1	1
Histotype		
ductal	45	42
lobular	0	8
others	1	8

**Abbreviations**: ER = estrogen receptor; PgR = progesterone receptor; HER2 = human epidermal growth factor receptor-2; CISH = chromogenic in situ hybridization; FISH = fluorescence in situ hybridization.

**Figure 1:**
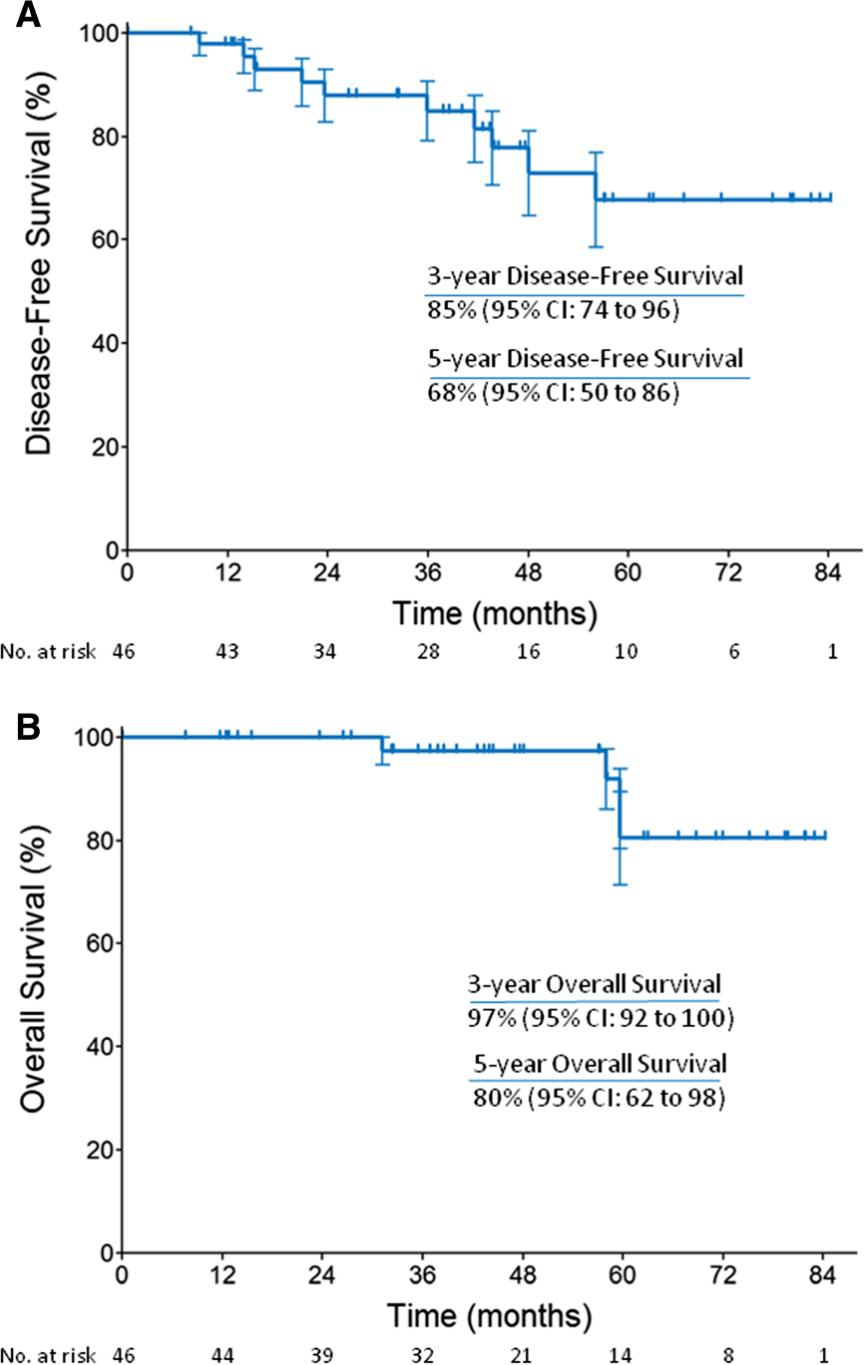
**Disease-free survival and Overall survival. A**. Disease-free survival and **B**. Overall survival in the HER2-positive group.

Clinical response data for HER2-positive patients are summarized in Table 2. Twenty-three pCR (50%) were achieved, with residual ductal carcinoma *in situ* in 7 patients. By intent-to-treat analysis after median follow-up of 42.9 months (7.5-84.2 months), 10 primary outcome events were reported, whereas the median DFS was not reached. Also the median OS was not reached after median follow-up time of 47.3 months (7.5-84.2 months) (Figure 1). At the end of treatment, all patients underwent surgery. Breast conserving surgery rate was 39.1% (18/46).

**Table 2:** **Response Rates to NC incorporating trastuzumab**

	N	PD	cPR	cCR	tpCR	bpCR
**Total**	46 (100%)	2 (4.4%)	23 (50%)	21 (45.6%)	23 (50%)	26 (56.5%)
**ER status**						
**Positive**	22 (47.8%)		13 (59%)	9 (41%)	10 (45.4%)	12 (54.5%)
**Negative**	24 (52.2%)	2 (8.3%)	10 (41.7%)	12 (50%)	13 (54.2%)	14 (58.3%)
**Stage distribution**						
**IIA**	6 (13%)	1(16.6%)	4 (66.8%)	1 (16.6%)	5 (83%)	5 (83%)
**IIB**	27 (58.7%)		13 (48%)	14 (52%)	11 (41%)	14 (52%)
**IIIA**	12 (26.1%)	1 (8.3%)	5 (41.7%)	6 (50%)	7 (58.3%)	7 (58.3%)
**IIIB**	1 (2.2%)		1 (100%)			

**Abbreviations**: PD = progression disease; cPR = clinical partial response; cCR = clinical complete response; tpCR = total pathological complete response; bpCR = breast pathological complete response.

### Safety data

The adverse events observed in the HER2-positive group are reported in Table 3. Three patients experienced grade 3 onychopathy and only one patient developed onychopathy that progressed to grade 4. One patient developed a peripheral neuropathy grade 3, whereas all other adverse events were generally mild. Because of onychopathy and peripheral neuropathy, 3 patients needed dose reductions. Of note, none of these patients developed symptomatic (grade 3/4) heart failure. Left ventricular dysfunctions were not reported.

**Table 3:** **Worst hematological and non-hematological toxic effects in 46** ***HER2-*** **positive BC patients**

Adverse events	G1	G2	G3	G4
Leucopenia	1	1		
Neutropenia	1		1	
Neutropenic fever				
Anaemia	5			
Thrombopenia				
Hyperthransaminasemia	2			
Alopecia	17	29		
Mucositis	7	2	1	
Nausea -Vomiting	10	1		
Epigastralgia	4			
Diarrhea	1	2		
Constipation	3	1		
Fatigue/weakness	5	2		
Peripheral neuropathy	13	15	1	
Onychopathy	6	12	3	1
Arthralgia - myalgia	5	1		
Oedema legs	8	1		
Skin toxicity	3	2		
Flush	2	6		
Epistaxis	3			
Left ventricular dysfunction	4			

### Serum cytokine profiling

**Figure 2:**
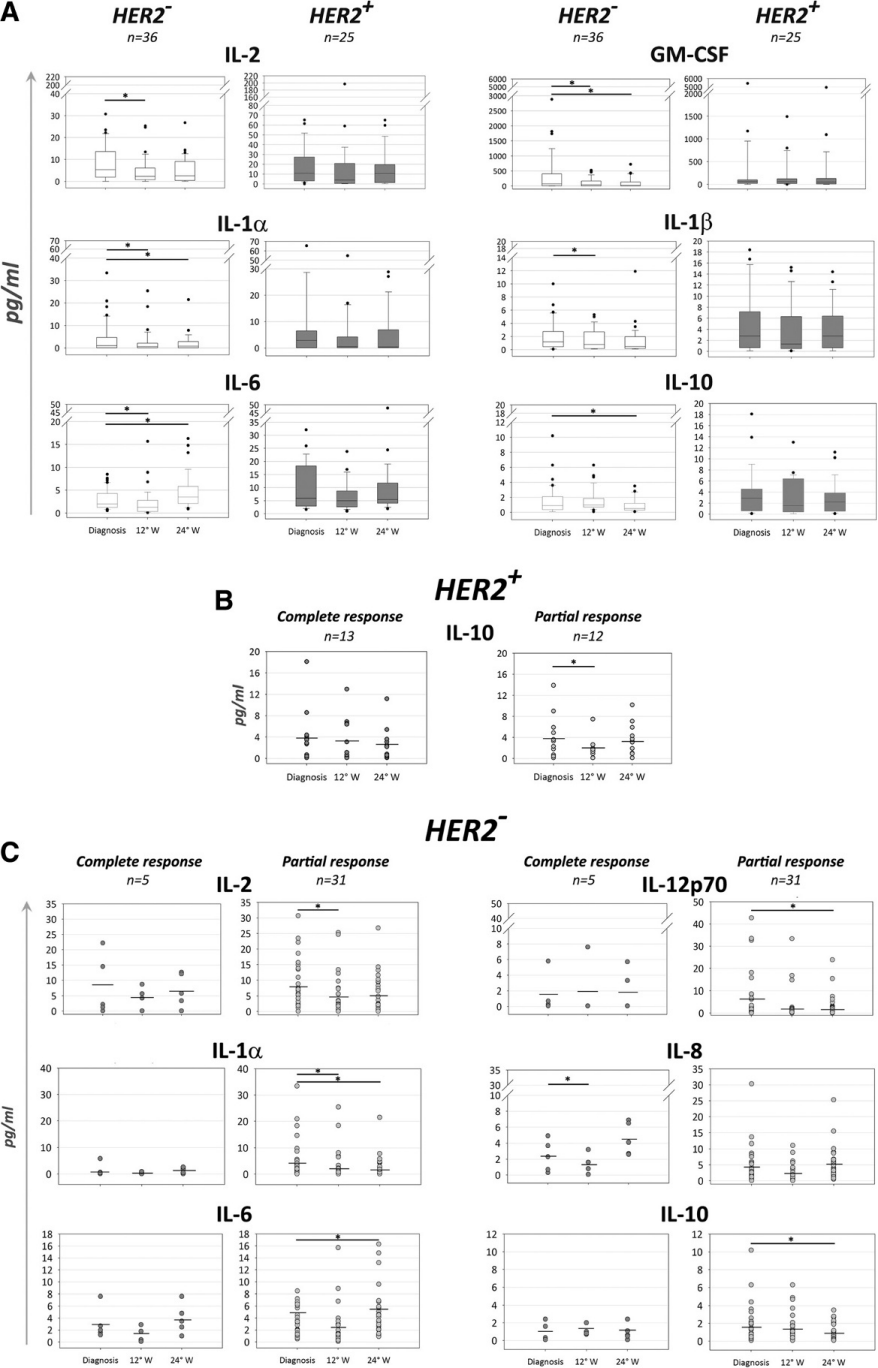
**Serum cytokine profile.** Interleukin (IL)-2, IL-12p70, IL-1α, IL-1β, IL-8, IL-6, IL10, and granulocyte-macrophage colony-stimulating factor (GM-CSF) levels were evaluated in serum samples from HER2-negative (n = 36) and HER2-positive (n = 25) patients at diagnosis, and at the 12° and 24° weeks (W) of NC treatment. **A**. Trend of cytokine levels throughout NC in the 2 groups of treatment. Box plots represent the median values, the 25th, and the 75th percentiles. **B**. IL-10 levels in HER2-positive patients achieving a complete (n = 13) or a partial (n = 12) pathological response. Each circle symbolizes the IL-10 concentration measured in each patient. The mean value is indicated. **C**. Cytokine levels in HER2-negative patients divided in individuals achieving a complete (n = 5) or a partial (n = 31) pathological response. Statistical analysis was performed with the Student’s t test; *P < 0.05. HER2, human epidermal growth factor receptor-2. HER2^+^, HER2-positive patients. HER2^-^, HER2-negative patients.

Serum levels of 10 different cytokines were evaluated at diagnosis, after 12 and 24 weeks in 25 HER2-positive and in 36 HER2-negative patients. No significant changes were highlighted in the HER2-positive group for any of the cytokines investigated throughout NC treatment, whereas significant alterations were observed in HER2-negative patients during ED chemotherapy (a decrease in IL-2, GM-CSF, IL-1α [*P* = 0.01], IL-1β [*P* = 0.03], and IL-6 amounts [*P* = 0.03] after 12 weeks, a decrease in the levels of GM-CSF, IL-1α [*P* = 0.01], and IL-10 [*P* = 0.04] after 24 weeks, and increase in IL-6 levels [*P* =0.02] at 24 weeks of NC) (Figure 2A).

Comparison of HER2-positive patients undergoing pCR with those achieving partial responses disclosed a significant decrease in IL-10 levels (*P* = 0.02) after 12 weeks of TP therapy only in partial responders (Figure 2B). Similarly, altered cytokine levels were more frequently observed in HER2-negative patients with partial pathological responses (a decrease in the levels of IL-2 and IL-1α [*P* = 0.02] at 12 weeks, a decrease in IL-12p70 [*P* = 0.05], IL-1α [*P* = 0.01], IL-10 [*P* = 0.03] amounts at 24 weeks, and an increase in IL-6 levels [*P* = 0.03] after 24 weeks of NC) (Figure 2C). Patients achieving pCR after ED therapy revealed a significant decrease in IL-8 levels (*P* = 0.02) at week 12 (Figure 2C).

### Integrated immunomonitoring of Trastuzumab-mediated ADCC activity

Thirty-seven HER2-positive patients were genotyped for the FcγRIIIa-158 valine (V)/phenylalanine (F), the FcγRIIa-131 histidine (H)/arginine (R), and the FcγRIIb-187 isoleucine (I)/threonine (T) polymorphisms. No significant correlations with pCR were found for any of the polymorphisms analyzed (not shown).

**Figure 3:**
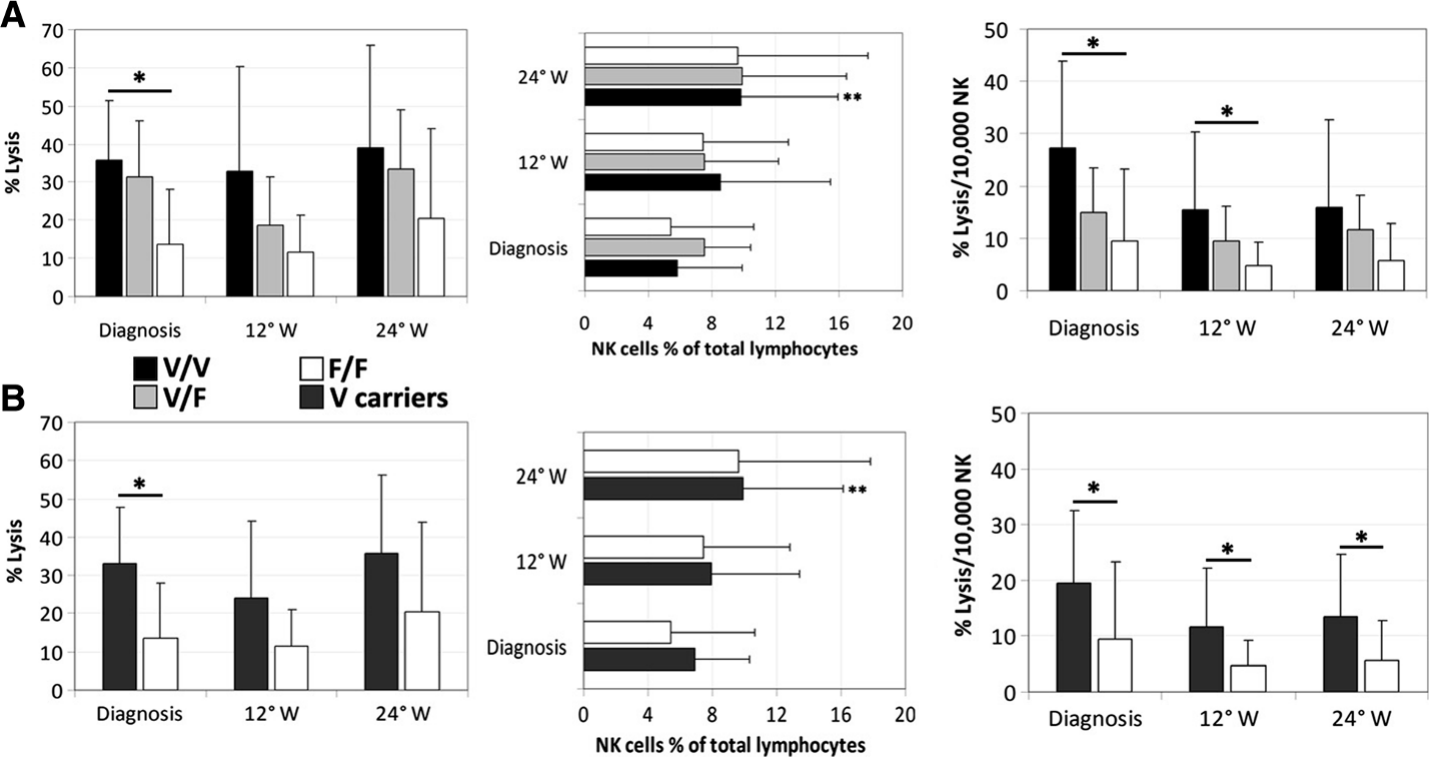
**Monitoring of Trastuzumab-mediated ADCC activity in HER2-positive patients according to the FcγRIIIa polymorphism.** Representative data obtained against the HER2-overexpressing cell line MDA-MB-453 using patients’ PBMCs collected at diagnosis, and at the 12° and 24° weeks (W) of NC treatment. **A**. Data were classified by the V/V (n = 9), V/F (n = 17), and F/F (n = 10) genotypes of the FcγRIIIa polymorphism. Statistical analysis was performed with the Kruskal-Wallis test. **B**. Analysis was performed comparing V carriers (n = 26) with F/F individuals, through the Wilcoxon test. Graphs on the left show the absolute percentage of lysis measured at a 30:1 Effector:Target ratio. Natural Killer (NK) cells were identified in flow cytometry as CD3-CD16 + CD56dim lymphocytes. Histogram plots on the right display normalized lysis calculated for 10,000 NK cells. Data are represented as means and standard deviations. The Student t test was employed for NK cells percentage variations. *Chi-square or |Z| < 0.05. **P < 0.05. HER2, human epidermal growth factor receptor-2.

Assessment of the ability of patients’ PBMCs to mediate trastuzumab-mediated ADCC showed a gradient of efficiency according to the FcγRIIIa-158 genotypes, with V/V > V/F > F/F at all time points investigated and a significant difference observed at diagnosis (*P* = 0.01; Figure 3A). The gradient in lysis efficiency was still observed after normalization for the number of NK cells, the main ADCC mediators [11], with significant differences both at diagnosis (*P* = 0.01) and at week 12 (*P* = 0.04; Figure 3A). Notably, the number of circulating NK cells increased along the NC treatment, with a difference that was statistically significant after 24 weeks only in V/V patients (*P* = 0.01; Figure 3A). V carriers (V/V + V/F) had effectors eliciting a higher percentage of ADCC lysis at diagnosis compared to F/F patients (*P* < 0.01), showed a significant increase in the number of circulating NK cells after 24 weeks (*P* = 0.01), and maintained a higher ADCC efficacy (normalized lysis) at diagnosis and throughout NC (*P* = 0.01 at diagnosis; *P* = 0.02 at week 12; *P* = 0.05 at week 24) with respect to patients carrying the unfavourable genotype (Figure 3B).

## Discussion

This study confirms that an anthracycline-free NC regimen for locally advanced HER2-positive BC patients can be safely used without compromising the clinical outcome, provided that trastuzumab is given concurrently with a weekly taxane. The 50% pCR rate observed in our study is highly promising considering that similar outcomes have been previously obtained only with anthracycline-containing regimens [1, 3, 4]. Moreover, between HER2-negative and HER2-positive BC, a difference of 36% in pCR rate was observed. These results are in line with those shown in the NOAH trial in which a difference of 26% in pCR rate between HER2-positive (43%) and HER2-negative (17%) BC was detected [3, 4].

Our findings are particularly relevant in the light of the recent demonstration that, in selected groups of patients, chemotherapy could be entirely avoided. In fact, in the NeoSphere study, Trastuzumab/Pertuzumab combination yielded a 16.8% pCR rate, which increased up to 29% in the ER/PgR-negative subgroup [18]. Moreover, in the NeoALTTO study, the double HER2 block induced by Trastuzumab/Lapatinib combination showed significant clinical response rates 6 weeks before starting chemotherapy [16]. Our results provide also possible indications regarding the chemotherapeutic backbone associated with trastuzumab, a still open issue in relation to efficacy and safety, especially considering long-term outcomes. In fact, while pCR rate obtained by trastuzumab in combination with paclitaxel administered every three-weeks was only 18% in stage II/III BC [19], our results are consistent with a more synergic activity of trastuzumab when associated with paclitaxel in the weekly schedule [20].

Besides showing clinical efficacy, our anthracycline-free NC regimen was not burdened by severe cardiac toxicity. In fact, no patients developed symptomatic congestive heart failure and only 4 patients showed a LVEF grade 1 reduction. Although limited by the small number of patients enrolled and by median follow up with a wide confidence interval, we report an 8.6% rate (4 out of 46) of asymptomatic cardiotoxicity, which is in line with previous studies, and a lower incidence of symptomatic cardiac events than expected (0.74-1.9). In the NOAH trial, the concurrent use of doxorubicin and trastuzumab was associated with an incidence of cardiac events lower (<2%) than expected on the basis of adjuvant trials results with trastuzumab and anthracyclines [3, 4]. Indeed, the duration of trastuzumab and the limited exposure to epirubicin concomitantly with trastuzumab could have influenced the cardiac safety, as demonstrated by previous findings reporting no clinical congestive heart failure [1]. In this context, our results indicate that the trastuzumab-paclitaxel combination represents a well-tolerated and heart-safe regimen, consistently with previous studies on anthracycline-free adjuvant regimens in HER2-positive BC [21]. In addition, our data are in keeping with those recently reported at the 2013 San Antonio Breast Cancer Symposium with the same regimen in 406 node-negative HER2-positive patients treated in the adjuvant setting [22]. The impressive results obtained without anthracyclines, at least in a selected population of patients, may become “practice-changing” in the next few years.

Achievement of pCR in patients with HER2-overexpressing BC was associated with better distant metastasis-free survival, recurrence-free survival, DFS and OS [15, 23, 24]. In our study, only 3 of the 10 HER2-positive patients showing a recurrence had previously achieved a pCR. Extended follow-up is needed to reach the median DFS and OS, and to draw definite conclusions. In this respect, while in the absence of trastuzumab NC including anthracyclines still induces superior outcomes [25], a recent paper by Guiu and colleagues [26] reported competitive results in terms of DFS and OS in locally advanced HER2-positive BC patients treated with anthracycline-free trastuzumab-based NC.

Immune profiling of our series of patients with HER2-overexpressing BC demonstrated a retained immune proficiency at diagnosis [13]. Consistently, here we show that the same patients had unaltered cytokine levels during TP therapy, suggesting that the clinical benefit achieved by trastuzumab and taxanes may partially rely on the immune-mediated mechanisms of these drugs [27]. Conversely, in the HER2-negative cohort, which is different from the HER2-positive arm for the biology of the tumor, the NC treatment, and also for the immune profile at diagnosis [13], we observed changes in IL-1α, IL-1β, IL-2, IL-6, and GM-CSF levels already at week 12 of ED treatment. Intriguingly, the only cytokine abnormality observed in the HER2-positive group, a decrease in IL-10 levels, was restricted to patients undergoing partial pathological responses. IL-10 is a pleiotropic cytokine, which may favour NK cell-dependent lysis through MHC class-I down-regulation on tumor cells [28, 29]. This potential effect of IL-10 may synergize with the ability of taxanes to increase NK cell activity [6], and could support a correlation between pCR and maintained IL-10 levels.

NK cells are key factors for an efficient trastuzumab-mediated ADCC [27]. We noticed, at diagnosis, different ADCC efficacy among patients carrying the V/V, V/F, or F/F genotypes of the activating FcγRIIIa receptor. This polymorphism correlated with improved clinical responses observed after trastuzumab in some instances but not in others [9, 30]. Notably, comparing the 3 FcγRIIIa-158 genotypes, we observed different levels of normalized ADCC at diagnosis, suggesting the primary role of NK cells in mediating trastuzumab efficacy and the functional relevance of the FcγRIIIa-158 polymorphism. We also noticed that patients carrying the favourable V allele maintained a high ADCC efficiency (normalized lysis) throughout the NC treatment, and showed a significant increase in NK cell percentage after 24 weeks of therapy. These findings are consistent with a synergistic activity between trastuzumab and taxanes, partially dependent on immune mechanisms [8, 31], and are in keeping with the reported ability of this drug combination to promote NK cell recruitment and activation [32]. Conversely, the concurrent chemotherapy with anthracyclines or alkylating agents seemed to diminish the influence of the FcγRIIIa genotype on patients’ outcome when treated with trastuzumab [30]. Intriguingly, previous findings demonstrating an association between trastuzumab-mediated ADCC and a favourable outcome in operable HER2-overexpressing BC patients supported the importance of an uncompromised immune function in patients with early-stage tumors [6, 11]. With this phase II study, we demonstrated that patients with locally advanced HER2-overexpressing BC still maintain an immune proficiency that could be exploited by drugs acting synergistically through immune-mediating mechanisms.

## Conclusions

In conclusion, the present phase II study reports a high rate of pCR in locally advanced HER2 overexpressing BC patients treated with a NC regimen excluding anthracyclines and combining trastuzumab and paclitaxel in a weekly schedule. This therapeutic choice is characterized by a low toxicity as demonstrated by the reduced cardiotoxicity and the maintained immune proficiency in BC patients. Our results are consistent with the possibility that the synergy between the immunomodulating properties of the drugs used in this trial and the retained immune competence observed in BC patients may favor the induction of pCR. In perspective, longer follow-up of our cohort of patients will provide evidence supporting the possible correlation between the high pCR rate obtained with anthracycline-free NC regimen and both a longer disease-free interval, and a definite low risk of cardiotoxicity. Furthermore, immunomonitoring of follow-up patients will reveal whether the immunological parameters identified in this study may be regarded as biomarkers potentially able to predict the clinical outcome of locally advanced HER2-overexpressing BC patients treated with regimens devoid of anthracyclines.

## Authors’ information

Diana Crivellari and Riccardo Dolcetti shared senior authorship.

## Abbreviations

ADCC: Antibody-Dependent Cell Cytotoxicity
BC: Breast Cancer
BCT: Breast Conservation Therapy
DFS: Disease-Free Survival
ED: Epirubicin Docetaxel
FBS: Fetal Bovine Serum
GM-CSF: Granulocyte Macrophage Colony-Stimulating Factor
HER2: Human Epidermal growth factor Receptor 2
IHC: Immunohistochemistry
IL: Interleukin
LVEF: Left Ventricular Ejection Fraction
MHC: Major Histocompatibility Complex
NC: Neoadjuvant Chemotherapy
NK: Natural Killer
ORR: Objective Response Rate
OS: Overall Survival
PBMCs: Peripheral Blood Mononuclear Cells
pCR: pathological Complete Response
PCR: Polymerase Chain Reaction
RECIST: Response Evaluation Criteria In Solid Tumors
TGF: Transforming Growth Factor
TNF: Tumor Necrosis Factor
TP: Trastuzumab Paclitaxel
UNL: Upper Normal Limit.
